# Mutational analysis of the essential lipopolysaccharide-transport protein LptH of *Pseudomonas aeruginosa* to uncover critical oligomerization sites

**DOI:** 10.1038/s41598-020-68054-7

**Published:** 2020-07-09

**Authors:** Romina Scala, Adele Di Matteo, Antonio Coluccia, Alessandra Lo Sciuto, Luca Federici, Carlo Travaglini-Allocatelli, Paolo Visca, Romano Silvestri, Francesco Imperi

**Affiliations:** 1 Department of Biology and Biotechnology “Charles Darwin”, Sapienza University of Rome, Rome, Italy; 2 Institute of Molecular Biology and Pathology, CNR, Rome, Italy; 3 Department of Drug Chemistry and Technologies, Sapienza University of Rome, Rome, Italy; 4 Laboratory affiliated to Istituto Pasteur Italia – Fondazione Cenci Bolognetti, Rome, Italy; 5 Department of Science, Roma Tre University, Viale G. Marconi 446, 00146 Rome, Italy; 6 Department of Medical, Oral and Biotechnological Science and C.A.S.T. Center for Advanced Studies and Technology, “G. d’Annunzio” University of Chieti-Pescara, Chieti, Italy; 7 Department of Biochemical Sciences “A. Rossi Fanelli”, Sapienza University of Rome, Rome, Italy

**Keywords:** Bacteria, Bacteriology

## Abstract

Lipopolysaccharide (LPS) is a critical component of the outer membrane (OM) of many Gram-negative bacteria. LPS is translocated to the OM by the LPS transport (Lpt) system. In the human pathogen *Pseudomonas aeruginosa*, the periplasmic Lpt component, LptH, is essential for LPS transport, planktonic and biofilm growth, OM stability and infectivity. LptH has been proposed to oligomerize and form a protein bridge that accommodates LPS during transport. Based on the known LptH crystal structure, here we predicted by in silico modeling five different sites likely involved in LptH oligomerization. The relevance of these sites for LptH activity was verified through plasmid-mediated expression of site-specific mutant proteins in a *P. aeruginosa lptH* conditional mutant. Complementation and protein expression analyses provided evidence that all mutated sites are important for LptH activity in vivo. It was observed that the *lptH* conditional mutant overcomes the lethality of nonfunctional *lptH* variants through RecA-mediated homologous recombination between the wild-type *lptH* gene in the genome and mutated copies in the plasmid. Finally, biochemical assays on purified recombinant proteins showed that some LptH variants are indeed specifically impaired in oligomerization, while others appear to have defects in protein folding and/or stability.

## Introduction

The cell envelope of diderm (Gram-negative) bacteria consists of two concentric membranes, the inner (IM) and outer membrane (OM), which confine an aqueous compartment, the periplasmic space, in which a thin layer of peptidoglycan is embedded. While the IM is a typical phospholipids bilayer, the OM of most diderm bacteria is an asymmetric membrane composed of lipopolysaccharide (LPS) and phospholipids in the outer and inner leaflets, respectively^1^. LPS is a negatively charged glycolipid that forms a tightly packed layer at the cell surface. The LPS layer is important for the structural stability of the OM, and provides an effective permeability barrier to the entry of potentially noxious compounds^2^.

**Figure 1:**
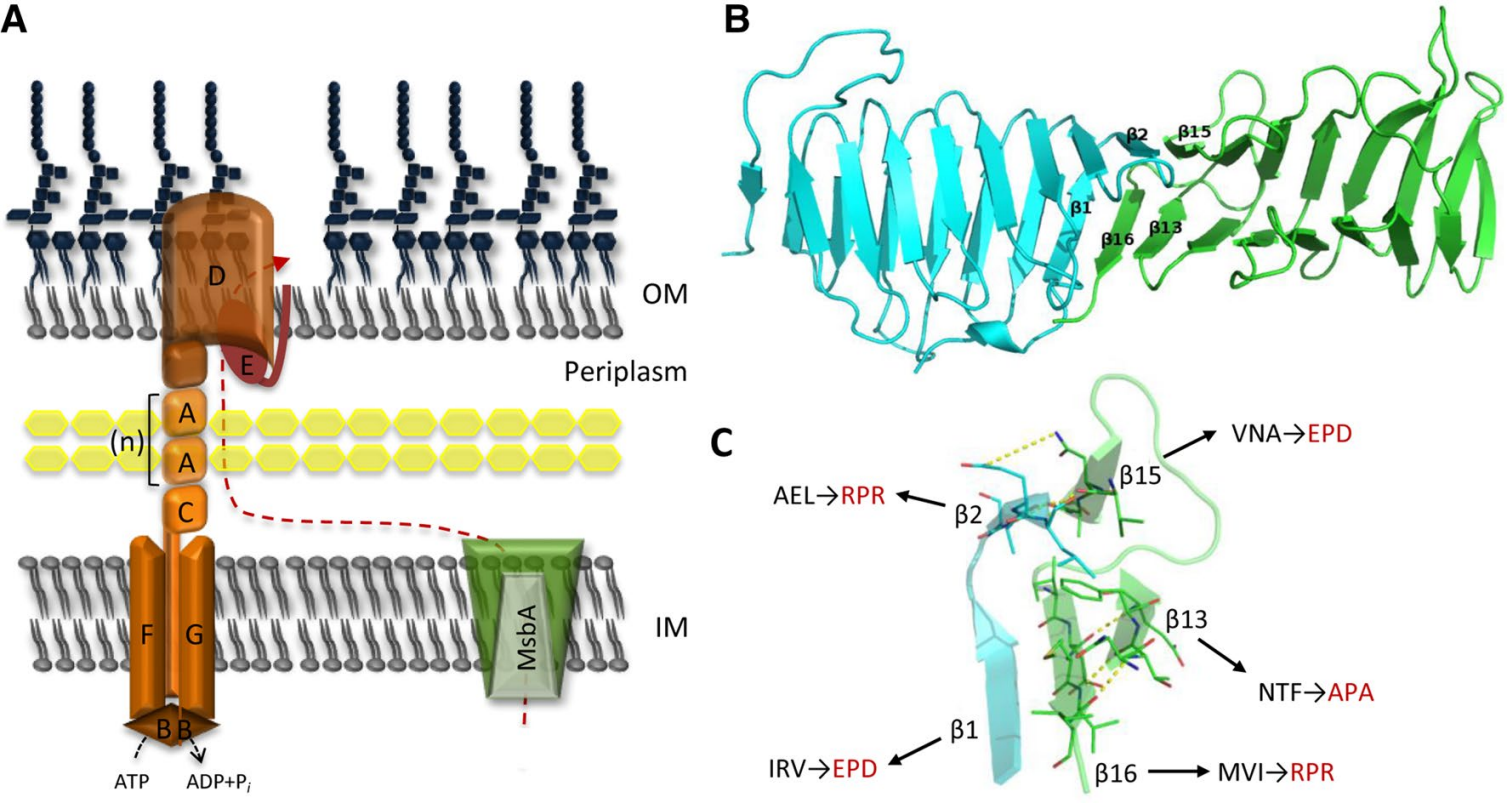
(**A**) Current model for the LPS transport pathway of *E. coli*. LPS is translocated to the periplasmic side of the IM by the ABC transporter MsbA, extracted from the IM in an ATP-dependent manner by the IM complex LptB_2_FG, and pushed through the hydrophobic cavity of the protein bridge formed by the β-jellyroll domains of LptC, LptA, and the N-terminal region of LptD. The number of LptA monomers in the bridge is unclear. The C-terminal domain of LptD forms the β-barrel that, assisted by the OM lipoprotein LptE, inserts LPS into the OM. (**B**) Model of the head-to-tail LptH dimer obtained by molecular dynamic simulation. The monomers are reported as cyan and green cartoons for the head and tail protomers, respectively. (**C**) Close view of strands contacts β1–β16, β2–β15 and β13–β16. H-bonds are reported as yellow-dot lines. Arrows highlight the amino acid residues that have been either deleted or replaced in LptH mutant variants: wild-type residues are in black, mutant residues present in LptH variants β1mut, β13mut, β15mut and β16mut are in red (Table 1)*.* The β2mut variant was not obtained and, thus, not tested in this work (see text for details).

LPS is synthesized in the cytoplasm, matured in the periplasm and translocated to the OM by the Lpt (Lipopolysaccharide transport) system that, in the model organism *Escherichia coli*, is composed of seven essential proteins (LptABCDEFG). The Lpt protein complex spans the entire cell envelope and consists of two sub-assemblies, LptB_2_CFG at the IM and LptDE at the OM, connected by the periplasmic protein LptA^3–5^. LptB_2_FG is an ATP-binding cassette (ABC) transporter that, in association with the bitopic protein LptC, powers LPS transport to the cell surface, while the β-barrel protein LptD and the lipoprotein LptE constitute the OM translocon that inserts LPS into the outer leaflet of the OM^3–5^ (Fig. 1A).

Interestingly, Lpt components with a periplasmic localization (i.e., LptA) or having a periplasmic domain (LptC, LptF, LptG and LptD) share a very similar β-jellyroll architecture^6^. Photo-crosslinking, co-purification and structural studies support the idea that LptC, LptA and the N-terminal region of LptD interact by means of their homologous β-jellyroll domains and form a protein bridge that provides a continuous hydrophobic groove to accommodate the lipid A moiety of LPS during translocation across the aqueous periplasmic environment^7,8^. According to the recently proposed model, LPS moves from the IM to the OM through a series of energy-dependent steps, powered by ATP hydrolysis, that sequentially push LPS molecules through the periplasmic protein bridge in a continuous flow^8,9^.

The number of LptA monomers in the periplasmic bridge is still debated. The presence of two or more LptA monomers is supported by the propensity of LptA to form head-to-tail oligomers in vitro^10–12^, and LptA oligomerization was also observed in vivo^7^. However, it has been recently demonstrated that a truncated LptA variant, that lacks the entire C-terminal region and that is not able to oligomerize, can partially support *E. coli* growth^13^, suggesting the LptA oligomerization could not be strictly required for LPS transport.

The structure of the LptA ortholog of the human pathogen *Pseudomonas aeruginosa*, named LptH, has been recently solved. Despite the poor sequence homology, LptH shares identical β-jellyroll fold with the *E. coli* counterpart^14^. However, differently from *E. coli* LptA, that can form long head-to-tail oligomers in solution also at low concentration^11^, LptH mainly exists as a dimer in solution^14,15^. However, a continuous fiber-like arrangement of LptH protomers has been observed in crystal lattice and just before the crystallization trials set up^14^.

Through conditional mutagenesis, we have previously confirmed the essentiality of LptH for *P. aeruginosa* cell survival, growth, biofilm formation, antibiotic resistance and infectivity in different animal models^16,17^. These findings highlight LptH as a promising molecular target for the design of novel anti-*P. aeruginosa* drugs. This study was aimed at identifying and validating the LptH residues that are involved in protein dimerization, in order to verify whether oligomerization sites are actually important for LptH activity and, thus, to propose LptH oligomerization sites as potential drug development targets.

## Results

### Prediction of LptH sites involved in oligomerization by in silico modelling

The Lpt machinery component LptH is predicted to oligomerize to form a protein bridge across the periplasm that allows the flow of LPS from the IM to the OM. The three-dimensional structure of LptH consists of a 16 antiparallel β-strands (β1–β16) folded into a slightly twisted β-jellyroll. It has been proposed that LptH oligomerizes in a head-to-tail fashion, as observed in the *E. coli* orthologue LptA^14^.

Here, the structure of the head-to-tail LptH dimer was drawn by superimposition with the *E. coli* LptA dimer structure^10^. Indeed, despite their low sequence identities, these proteins share very close folding^14^. The obtained model was submitted to molecular dynamic simulation (100 ns) to resolve clashes and to identify the most robust interactions between the two lobes of the dimer. The trajectory inspection predicted a series of stable contacts between the two monomers (Fig. 1B). According to the model, the strands implicated in dimerization of the LptH monomers are the β1 (IRVQA) and β2 (SAEL) of the head monomer and β15 (IVNAG) and β16 (DMVIQ) of the tail monomer. Each of these strands was involved in extensive hydrophobic contacts with the other three strands. Furthermore, each strand forms H-bonds with the adjacent strand (β1 with β6 and β2 with β15). These lateral H-bonds were peculiar for the β-jellyroll fold^18^. Furthermore, we observed a H-bond between the polar side chains of Arg^34^ (β1) and Asp^165^ (β16), and another one between the polar side chains of Glu^41^ (β2) and Asn^148^ (β15) (Fig. 1C). Trajectory analyses suggested that also the β13 strand (NTFEG) might be important for dimerization. The bulkier Phe^133^ (β13) is indeed predicted to form hydrophobic contacts with the residues of the β1, β15 and β16 strands (Fig. 1C), that could be involved in dimer stabilization. Furthermore, Phe^133^ (β13) is at bond distance from Tyr^51^ (β3), likely allowing aromatic interaction (Fig. 1C). This weak interaction could drive a “closed conformation” of the dimer when LPS is not bound. A similar behaviour was also predicted for Tyr^91^ (β8), Tyr^111^ (β10) and Tyr^140^ (β14), which could interact each other to stabilize the “closed conformation” of the dimer (data not shown).

### In vivo functional assessment of LptH variants

To verify the relevance of the putative oligomerization sites identified by molecular modelling, we introduced in the LptH protein sequence either a 3-amino acid deletion or a 3-amino acid substitution for each interacting β-strand, according to the scheme reported in Fig. 1C. The amino acid substitutions were arbitrarily chosen in order to destabilize the β-strand secondary structure, by introducing a proline residue, and to alter the biochemical features of each site, by replacing the wild-type amino acids with amino acids characterized by opposite chemical properties. Each mutant was named based on the mutagenized β-strand or the type of mutation (for instance, variants carrying a 3-amino acid deletion and a 3-amino acid substitution in β1 were named β1del and β1mut, respectively) (Table 1).

**Table 1:** Bacterial strains and plasmids used in this work.

Strain	Relevant characteristics	Source or reference
***E. coli***		
S17.1λ*pir*	*thi pro hsdR hsdM*^+^ *recA* RP4-2-Tc::Mu-Km::Tn7*λpir,* Sm^R^	^39^
NEB 5-alpha′	*fhuA2 (argF-lacZ)U169 phoA glnV44 80 (lacZ)M15 gyrA96 recA1 relA1 endA1 thi-1 hsdR17*, Nal^R^	New England Biolabs
BL21 (DE3)	F^−^*ompT hsdS*_B_(r_B_^−^m_B_^−^) *gal dcm* (DE3)	^40^
***P. aeruginosa***		
PAO1 (ATCC15692)	Prototroph	American Type Culture Collection
PAO1 Δ*recA*	PAO1 with an in-frame deletion of the *recA* coding sequence	This work
PAO1 *araC*P_BAD_*lptH* Δ*lptH*	PAO1 with an arabinose-inducible copy of *lptH* (PA4460) inserted into the *attB* neutral site and an in-frame deletion of the endogenous copy of *lptH*	^16^
PAO1 *araC*P_BAD_*lptH* Δ*lptH* Δ*recA*	PAO1 *araC*P_BAD_*lptH* Δ*lptH* with an in-frame deletion of the *recA* coding sequence	This work
**Plasmid**		
pBluescript II (pBS)	Cloning and sequencing vector, Ap^R^	Stratagene
pBS *lptH*	pBS derivative carrying the wild-type *lptH* gene	This work
pBS *lptH*_β16del	pBS derivative carrying *lptH* with a 9-bp deletion involving codons 166–168	This work
pBS *lptH*_β16mut	pBS derivative carrying *lptH* with a 9-bp substitution in codons 166–168 leading to the mutation MVI → RPR	This work
pBS *lptH*_β13del	pBS derivative carrying *lptH* with a 9-bp deletion involving codons 131–133	This work
pBS *lptH*_β13mut	pBS derivative carrying *lptH* with a 9-bp substitution in codons 131–133 leading to the mutation NTF → APA	This work
pBS *lptH*_β1del	pBS derivative carrying *lptH* with a 9-bp deletion involving codons 33–35	This work
pBS *lptH*_β1mut	pBS derivative carrying *lptH* with a 9-bp substitution in codons 33–35 leading to the mutation IRV → EPD	This work
pBS *lptH*_β2del	pBS derivative carrying *lptH* with a 9-bp deletion involving codons 40–42	This work
pBS *lptH*_β15del	pBS derivative carrying *lptH* with a 9-bp deletion involving codons 147–149	This work
pBS *lptH*_β15mut	pBS derivative carrying *lptH* with a 9-bp substitution in codons 147–149 leading to the mutation VNA → EPD	This work
pME6032	Vector for IPTG-inducible expression in *P. aeruginosa*, *lacI*^Q^, Tc^R^	^36^
pME *lptH*	pME6032 derivative carrying an IPTG-inducible copy of *lptH*	This work
pME *lptH*_β16del	pME6032 derivative carrying an IPTG-inducible copy of *lptH*_β16del	This work
pME *lptH*_β16mut	pME6032 derivative carrying an IPTG-inducible copy of *lptH*_β16mut	This work
pME *lptH*_β13del	pME6032 derivative carrying an IPTG-inducible copy of *lptH*_β13del	This work
pME *lptH*_β13mut	pME6032 derivative carrying an IPTG-inducible copy of *lptH*_β13mut	This work
pME *lptH*_β1del	pME6032 derivative carrying an IPTG-inducible copy of *lptH*_β1del	This work
pME *lptH*_β1mut	pME6032 derivative carrying an IPTG-inducible copy of *lptH*_β1mut	This work
pME *lptH*_β2del	pME6032 derivative carrying an IPTG-inducible copy of *lptH*_β2del	This work
pME *lptH*_β15del	pME6032 derivative carrying an IPTG-inducible copy of *lptH*_β15del	This work
pME *lptH*_β15mut	pME6032 derivative carrying an IPTG-inducible copy of *lptH*_β15mut	This work
pDM4	Suicide vector in *P. aeruginosa*; *sacB, oriR6K*; Cm^R^	^37^
pDM4 Δ*recA*	pDM4 derivative for *recA* in-frame deletion	This work
pET28b	Plasmid for IPTG-inducible expression of proteins in the cytoplasm of *E. coli* BL21; Km^R^	Novagen
pET28b *lptH*	pET28b derivative carrying the *lptH* coding sequence, without its own signal sequence, fused in frame with the 6His coding sequence at the 5′ end	This work
pET28b *lptH_*β16del	pET28b derivative carrying the *lptH*_β16del coding sequence, without its own signal sequence, fused in frame with the 6His coding sequence at the 5′ end	This work
pET28b *lptH*_β16mut	pET28b derivative carrying the *lptH*_β16mut coding sequence, without its own signal sequence, fused in frame with the 6His coding sequence at the 5′ end	This work
pET28b *lptH_*β13del	pET28b derivative carrying the *lptH*_β13del coding sequence, without its own signal sequence, fused in frame with the 6His coding sequence at the 5′ end	This work
pET28b *lptH*_β13mut	pET28b derivative carrying the *lptH*_β13mut coding sequence, without its own signal sequence, fused in frame with the 6His coding sequence at the 5′ end	This work
pET28b *lptH_*β1del	pET28b derivative carrying the *lptH*_β1del coding sequence, without its own signal sequence, fused in frame with the 6His coding sequence at the 5′ end	This work
pET28b *lptH*_β1mut	pET28b derivative carrying the *lptH*_β1mut coding sequence, without its own signal sequence, fused in frame with the 6His coding sequence at the 5′ end	This work
pET28b *lptH_*β2del	pET28b derivative carrying the *lptH*_β2del coding sequence, without its own signal sequence, fused in frame with the 6His coding sequence at the 5′ end	This work
pET28b *lptH_*β15del	pET28b derivative carrying the *lptH*_β15del coding sequence, without its own signal sequence, fused in frame with the 6His coding sequence at the 5′ end	This work
pET28b *lptH*_β15mut	pET28b derivative carrying the *lptH*_β15mut coding sequence, without its own signal sequence, fused in frame with the 6His coding sequence at the 5′ end	This work

The wild-type and mutant alleles of *lptH* were individually cloned into the vector pME6032 under the control of an isopropyl-β-d-thiogalactoside (IPTG)-inducible promoter, in order to investigate whether the expression of the protein variants could restore the growth of an arabinose-dependent *P. aeruginosa lptH* conditional mutant^16^. Nine out of 10 mutant constructs of interest were successfully generated, while several attempts to obtain the *lptH_*β2mut variant failed. We therefore proceeded without this mutant, considering that the relevance of the corresponding site for LptH functionality could be evaluated through the β2del variant (Table 1).

**Figure 2:**
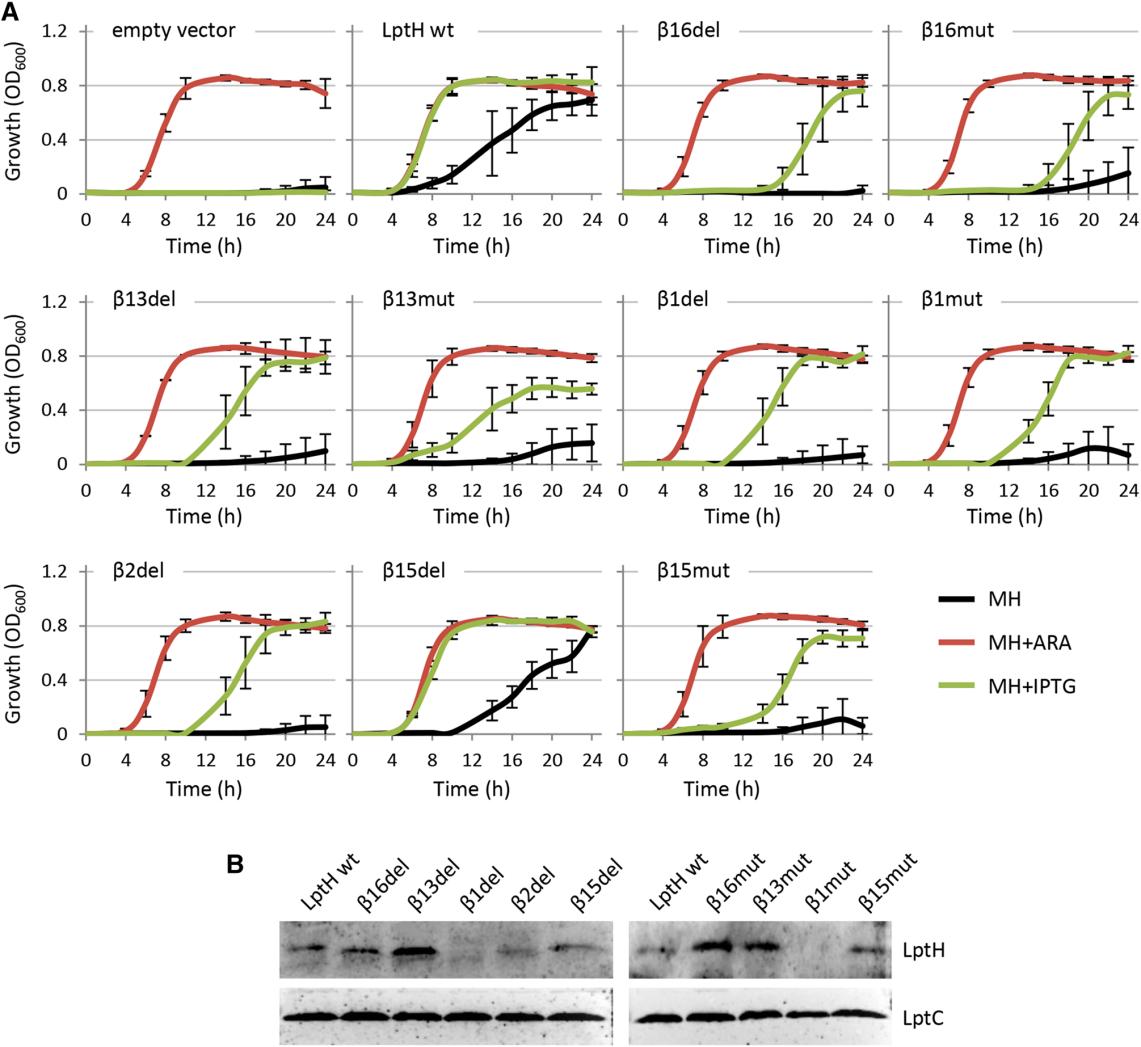
(**A**) Growth curves of the *P. aeruginosa lptH* conditional mutant carrying the empty plasmid pME6032, pME6032 with the wild-type gene *lptH* or pME6032 with different mutant variants in MH at 37 °C in microtiter plates in the absence (black lines) or in the presence of 0.5% arabinose (+ ARA; red lines) or 0.5 mM IPTG (+ IPTG; green lines). Growth was measured as OD_600_. Results are the mean (± SD) of three independent experiments, each one performed in triplicate. (**B**) Levels of LptH variants in the *P. aeruginosa lptH* conditional mutant carrying the different pME6032 derivatives cultured in MH supplemented with 0.5% arabinose and 0.5 mM IPTG, determined by Western blotting of whole-cell lysates (20 μg total proteins) with an anti-LptH polyclonal antibody (the empty vector used as negative control is shown in Figure S1). The housekeeping protein LptC was used as a loading control. Full-length blots are presented in Figure S5. Images are representative of three independent experiments which gave similar results.

Planktonic growth assays were performed to verify the ability of LptH variants to promote the growth of the *lptH* conditional mutant. The mutant carrying the empty vector or the plasmid expressing wild-type LptH (pME *lptH*) was used as negative or positive control, respectively. As expected, the *lptH* conditional mutant containing any of the constructs was able to grow in the presence of arabinose (Fig. 2A), which induces the expression of wild-type *lptH* in the genome (Table 1). Conversely, in the presence of IPTG, normal growth was only observed for cells carrying the constructs with wild-type *lptH* or the *lptH*_β15del variant (Fig. 2A). Surprisingly, cells expressing all the other LptH mutant variants did not grow for the first 10–14 h in the presence of IPTG, though they showed some delayed growth after a long lag phase. Such residual growth was, however, highly variable among different experimental replicates, as demonstrated by the high standard deviation values (Fig. 2A). A slightly different behaviour was observed for the *lptH*_β13mut construct, which did not show such long lag phase, although growth was strongly impaired with respect to the wild-type or the *lptH*_β15del construct during the entire growth curve (Fig. 2A). Finally, it should be noted that constructs that supported growth in the presence of IPTG (*lptH* and *lptH*_β15del) were also able to partially restore growth under non-inducing conditions (no IPTG, no arabinose). This is likely due to some leakiness of the IPTG-inducible promoter in *P. aeruginosa*^19^, especially from a multi-copy plasmid.

To verify that the growth defects of strains expressing the LptH mutant variants were not due to impaired expression/maturation of mutant proteins, the levels of the different LptH variants were evaluated by Western blot, using a custom polyclonal antibody raised against a LptH peptide conserved in all mutant variants (see Methods for details). Preliminary Western blot experiments showed that this antibody is not sensitive enough to detect LptH levels induced by arabinose in the *lptH* conditional mutant, while LptH was readily detected when expressed from the IPTG-inducible construct (Figure S1). This evidence supported the use of this anti-LptH antibody to specifically detect LptH variants from the IPTG-dependent multicopy plasmid. Most mutant proteins were expressed at levels comparable or even higher than those of wild-type LptH (Fig. 2B). In contrast, LptH_β2del showed slightly reduced expression and/or stability as compared to the wild-type protein, while LptH_β1del and LptH_β1mut levels were below the detection limit of the Western blot assay (Fig. 2B). However, since our anti-LptH antibody is not sensitive enough to detect physiological LptH levels (Figure S1), it can be possible that these two proteins are expressed at functionally relevant levels, and we therefore decided to include also these two mutant constructs in the subsequent analyses. Notably, whole cell lysates for Western blot analysis were obtained by culturing the *lptH* conditional mutant carrying the different constructs in the presence of both arabinose and IPTG, to sustain growth by inducing the chromosomal wild-type *lptH* allele and trigger expression of mutant *lptH* alleles from the plasmid. Interestingly, this analysis also revealed that none of the mutant proteins had detrimental effects on bacterial growth when co-expressed with wild-type LptH (Figure S2), denoting that the non-functional LptH variants do not exert dominant negative effects on functional (wild-type) LptH.

### Revertant mutants account for the residual growth of strains expressing non-functional LptH

**Figure 3:**
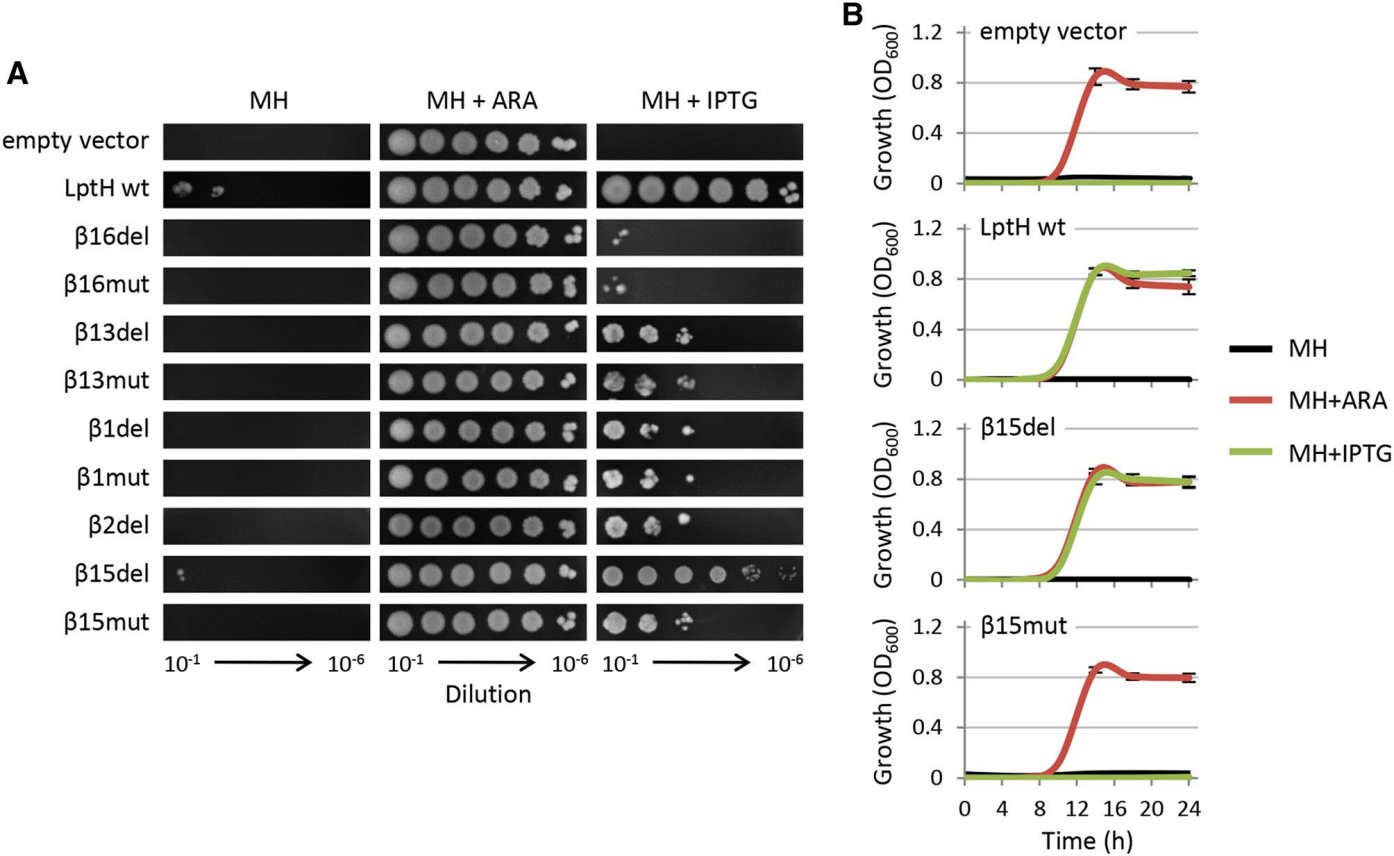
(**A**) Plating efficiency of the *P. aeruginosa lptH* conditional mutant carrying the pME6032 derivatives which express different LptH variants or the empty plasmid pME6032 on MH agar supplemented or not with 0.5% arabinose (+ ARA) or 0.5 mM IPTG (+ IPTG). Exponential phase cultures in MH with 0.5% ARA were normalized at OD_600_ = 1 in saline, and 5 μL of the 10^−1^ to 10^−6^ dilutions were spotted onto the plates, which were then incubated at 37 °C for 24 h. Pictures are representative of three independent experiments. (**B**) Growth curves of the *P. aeruginosa lptH* conditional mutant carrying the empty plasmid pME6032, pME6032 with wild-type *lptH* or pME6032 with selected mutant variants (β15del and β15mut) inoculated at a cell density of ca. 50–100 cells/mL in MH at 37 °C in microtiter plates in the absence (black lines) or in the presence of 0.5% arabinose (+ ARA; red lines) or 0.5 mM IPTG (+ IPTG; green lines). Growth was measured as OD_600_. Results are the mean (± SD) of three independent experiments, each one performed in quadruplicate. The remaining LptH mutant variants behaved the same as the β15mut protein (growth curves are shown in Figure S3).

To assess whether the delayed growth observed in the *lptH* conditional mutant expressing the LptH variants was an intrinsic feature of all cells in the population or was due to the presence of spontaneous mutations leading to phenotypic reversion, the plating efficiency of all strains was determined on agar plates in the presence and in the absence of arabinose or IPTG. As shown in Fig. 3A, the number of colonies obtained for the *lptH* conditional mutant carrying *lptH* and the *lptH*_β15del variant on plates supplemented with IPTG was comparable to that obtained on arabinose-containing plates, although *lptH*_β15del colonies were smaller in the presence of IPTG, suggestive of slightly decreased colony growth. The strains expressing all the other LptH variants showed strongly impaired plating efficiency, with relatively few colonies appearing on IPTG-containing plates (Fig. 3A). Table 2 shows the frequency of revertants obtained for each strain, calculated as the ratio of the number of colonies obtained on plates with or without IPTG to the number of colonies obtained on plates containing arabinose. Revertants frequencies ranged between 10^−4^ and 10^−3^ for *lptH* conditional mutant cells expressing the LptH β1, β2, β13 and β15 variants, while they were ten-fold lower (ca. 10^−5^) for cells expressing the LptH β16 variants (Table 2).

**Table 2:** Frequency of revertants for the *lptH* conditional mutant carrying the different pME6032 derivatives in MH agar with or without 0.5 mM IPTG.

Construct	Frequency of revertant mutants^a^	
		+ IPTG
pME6032	< 4.1 × 10^−7^	< 3.4 × 10^−7^
pME *lptH*	4.6 × 10^−5^	ca. 1
pME *lptH*_β16del	< 2.0 × 10^−7^	8.9 × 10^−6^
pME *lptH*_β16mut	< 3.2 × 10^−7^	1.4 × 10^−5^
pME *lptH*_β13del	< 2.9 × 10^−7^	7.0 × 10^−4^
pME *lptH*_β13mut	< 3.5 × 10^−7^	1.1 × 10^−3^
pME *lptH*_β1del	< 3.2 × 10^−7^	2.5 × 10^−4^
pME *lptH*_β1mut	< 3.7 × 10^−7^	1.1 × 10^−4^
pME *lptH*_β2del	< 2.4 × 10^−7^	3.5 × 10^−4^
pME *lptH*_β15del	2.1 × 10^−6^	ca. 1
pME *lptH*_β15mut	< 4.3 × 10^−7^	4.9 × 10^−4^

^a^Calculated from three independent assays performed in triplicate.

The above data strongly suggested that the significantly delayed growth in liquid cultures observed for the strains expressing non-functional LptH variants was due to the appearance of revertants. This hypothesis was indeed confirmed by monitoring planktonic growth of cultures inoculated with a number of cells (50–100) much lower than that expected to contain revertant mutants (based on the frequencies reported in Table 2). Under these conditions, planktonic growth was abolished in the presence of IPTG for strains expressing defective LptH variants, as well as for cells carrying the constructs with wild-type *lptH* or *lptH*_β15del in the absence of any inducers (Figs. 3B and S3). As expected, all strains grew well in the presence of arabinose, while strains expressing wild-type LptH or LptH_β15del were also able to grow in the presence of IPTG (Figs. 3B and S3), in line with the above results. Overall, these results demonstrate that the appearance of revertant mutants is responsible for (delayed) growth of strains expressing defective LptH variants.

### Homologous recombination is responsible for the emergence of revertants

The frequency of revertant mutants obtained in our assays (10^−3^–10^−5^) is much higher than that expected considering the spontaneous mutation rates observed in *P. aeruginosa* (10^−10^ and 10^−11^ per nucleotide per generation for base-pair and indel mutations, respectively)^20^. To rule out that the high number of revertants in the presence of IPTG could be due to an increased mutation rate in cells expressing defective LptH variants, that would ultimately increase the probability of emergence of “advantageous mutations” able to rescue growth, we compared the frequency of spontaneous resistant mutants for two different antibiotics (i.e., gentamicin and ofloxacin) between *lptH* conditional mutant cells expressing wild-type LptH or representative defective variants (β16del and β13mut), which showed the lowest and highest frequency of revertants in the plating efficiency assay, respectively (Table 2). The frequencies of resistance obtained for constructs expressing wild-type or defective LptH variants were comparable (Table S1), suggesting that genetic events other than spontaneous mutations could be responsible for the residual growth of strains carrying *lptH* variants.

We therefore hypothesized that homologous recombination between the arabinose-inducible copy of the *lptH* gene in the genome of the conditional mutant and the mutated copy carried by the plasmids could occur. This would result in the generation of plasmids carrying an IPTG-inducible wild-type copy of *lptH*, thus justifying the appearance of revertant colonies only on agar plates containing IPTG (Fig. 3A). To test this hypothesis, we sequenced the *lptH* coding sequence in plasmids extracted from revertant clones obtained on plates supplemented with IPTG. Notably, we found that all plasmids deriving from revertant colonies carried the wild-type copy of *lptH*. In contrast, the colonies of the conditional mutant harbouring the *lptH_*β15del construct, that showed a plating efficiency close to 1 (Table 2), still had the mutated *lptH* copy in the plasmid.

**Figure 4:**
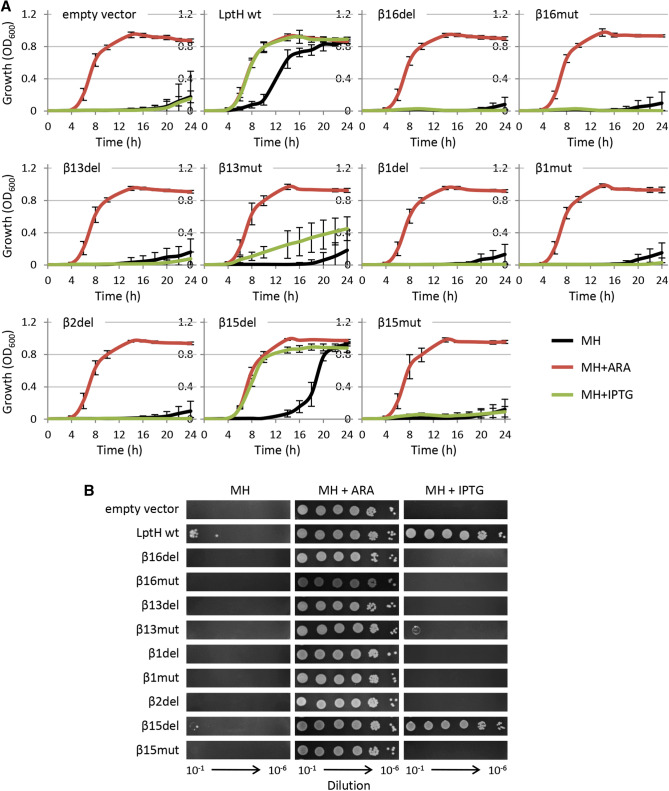
(**A**) Growth curves and (**B**) plating efficiency of the RecA-deficient *P. aeruginosa lptH* conditional mutant (PAO1 *araC*P_BAD_*lptH* Δ*lptH* Δ*recA*) carrying the empty plasmid pME6032, pME6032 with wild-type *lptH* or pME6032 with different mutant variants at 37 °C in MH in microtiter plates (panel **A**) or on MH agar (panel **B**) in the absence (black lines) or in the presence of 0.5% arabinose (+ ARA; red lines) or 0.5 mM IPTG (+ IPTG; green lines). Results are the mean (± SD) or are representative of three independent experiments, each performed in triplicate.

To confirm recombination as the molecular mechanism underlying the appearance of revertants, the *recA* gene, which is essential for homologous recombination^21^, was deleted in the *lptH* conditional mutant. The constructs carrying wild-type *lptH* or its mutant variants were then introduced in this RecA-deficient mutant. The constructs that were associated with delayed growth in the presence of IPTG in previous assays lost the ability to promote growth under the same culture condition (Fig. 4A). Moreover, no revertant mutants were observed on plates containing IPTG (Fig. 4B). This evidence corroborates the hypothesis that RecA-mediated homologous recombination with the wild-type genomic copy of *lptH* was responsible for the reversion of the IPTG-dependent mutant variants to the wild-type gene, which can obviously support the growth of the recombinant clones in the presence of IPTG. The only exception was the strain expressing the LptH_β13mut variant, which retained a partial ability to grow planktonically and showed some residual growth on IPTG-containing plates, although only at very high cell densities (Fig. 4). Finally, it should be noted that revertant mutants and residual planktonic growth in the absence of inducers (arabinose or IPTG) were observed for the wild-type *lptH* and *lptH*_β15del constructs also in the Δ*recA lptH* conditional mutant (Fig. 4). This indicates that, as reasonably expected, homologous recombination was not responsible for the growth of cells harbouring plasmids with the wild-type copy or a functional variant (β15del) of the *lptH* gene, implying that additional adaptive mechanisms might take place in cells expressing low levels of functional LptH protein. This issue, as well as the residual growth observed in cells expressing the LptH_β13mut variant, has not been further investigated in this work.

### Biochemical characterization of LptH mutant variants

**Figure 5:**
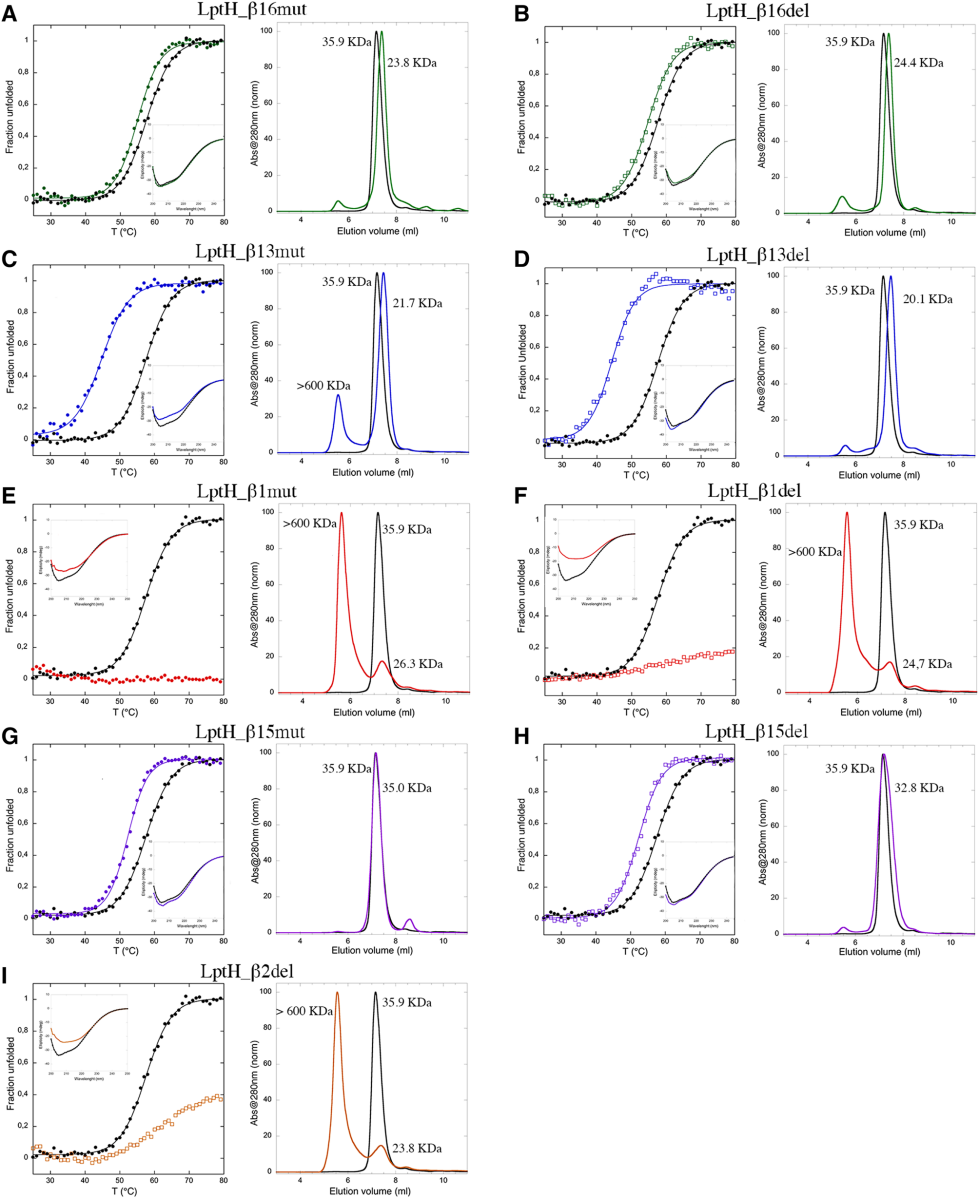
Folding and oligomerization properties of LptH variants. Thermal denaturation profile determined by far-UV CD at 220 nm (left panels) and gel filtration elution profile (right panels) of wild-type LptH (black lines) and the mutant variants (colored lines) LptH_β16mut (**A**), LptH_β16del (**B**), LptH_β13mut (**C**), LptH_β13del (**D**), LptH_β1mut (**E**), LptH_β1del (**F**), LptH_β15mut (**G**), LptH_β15del (**H**) and LptH_β2del (**I**). The inserts in the left panels show the relative CD spectra of the wild-type and variant proteins (black and colored lines, respectively).

To verify whether the defective LptH variants were actually impaired in oligomerization, N-terminally His-tagged variants of LptH and its mutants were expressed in the heterologous host *E. coli* and purified by Ni-affinity chromatography*.* Proteins were then characterized by size exclusion chromatography and CD spectroscopy. Far-UV CD thermal denaturation experiments showed a cooperative and reversible denaturation profile for the wild-type protein, with a T_m_ (57.5 °C) similar to that previously reported for LptH^14^ (Fig. 5). Gel filtration analysis showed that wild-type LptH elutes with a retention volume corresponding to an apparent molecular weight (MW) of ~ 36 kDa (Fig. 5), compatible with a dimeric state of the protein in solution.

The mutations introduced had different effects on LptH secondary structure, thermodynamic stability and oligomerization properties. LptH_β16mut and LptH_β16del showed secondary structure content and stability similar to the wild-type protein (T_m_ = 55.0 and 54.8 °C, respectively; Fig. 5A, B). Interestingly, these variants displayed a higher retention volume than the wild-type protein in the gel filtration assay, corresponding to an apparent MW of ~ 24 kDa (Fig. 5A, B). This observation suggests that variants with mutations in β16 have a lower dimerization propensity than the wild-type protein and that the monomeric state is prevalent in solution. LptH_β13mut and LptH_β13del were slightly destabilized, as evidenced by their lower T_m_ values (44.8 and 44.3 °C, respectively), but both showed a cooperative denaturation profile typical of a folded protein (Fig. 5C, D). Moreover, similarly to β16 variants, β13 mutants eluted with retention volumes higher than the wild-type protein, corresponding to apparent MWs of ~ 22 and 20 kDa for LptH_β13mut and LptH_β13del, respectively (Fig. 5C, D), suggestive of a predominant monomeric state. However, an additional elution peak corresponding to a MW > 600 kDa was observed for LptH_β13mut (Fig. 5C), implying that a fraction of the protein is present in a largely aggregated state. The elution profiles of variants LptH_β1mut and LptH_β1del were characterized by a main peak at low elution volumes, suggesting that most of the proteins were present as large aggregates with apparent MW > 600 kDa (Fig. 5E, F). The aggregation propensity of these LptH variants was also supported by thermal denaturation analysis. Indeed, a denaturation profile could not be obtained for these proteins (Fig. 5E, F), suggesting the presence of thermally stable aggregates. Moreover, the far-UV CD spectra obtained for both β1 variants are different from that obtained for wild-type LptH (Fig. 5E, F), further supporting the hypothesis that β1 variants are not properly folded. Similar results were obtained for LptH_β2del, showing non-detectable thermal denaturation, peculiar far-UV CD spectrum and gel filtration elution profile consistent with the presence of high MW aggregates (Fig. 5I). Variants LptH_β15mut and LptH_β15del showed a cooperative thermal denaturation profile typical of folded proteins, with a T_m_ value (52.3 °C) slightly lower than wild-type LptH (Fig. 5G, H). The apparent MWs calculated from gel filtration experiments (~ 35 and 33 kDa for LptH_β15mut and LptH_β15del, respectively) are consistent with a mainly dimeric state of the proteins in solution (Fig. 5G, H). It should be noted that, in the case of LptH_β15del, protein aggregation prevented us to test concentrations higher than 0.9 mg/mL.

### Modelling of the dimerization interface of selected LptH variants

To gain more insight into the inter-residues interactions that could promote the dimerization process in the LptH_β15mut and LptH_β15del variants, their dimers were modelled by molecular dynamics. The resulting trajectories were compared to those obtained for wild-type LptH and LptH_β16mut, used as controls for a dimerization-proficient and -deficient protein, respectively (Fig. 5).

**Figure 6:**
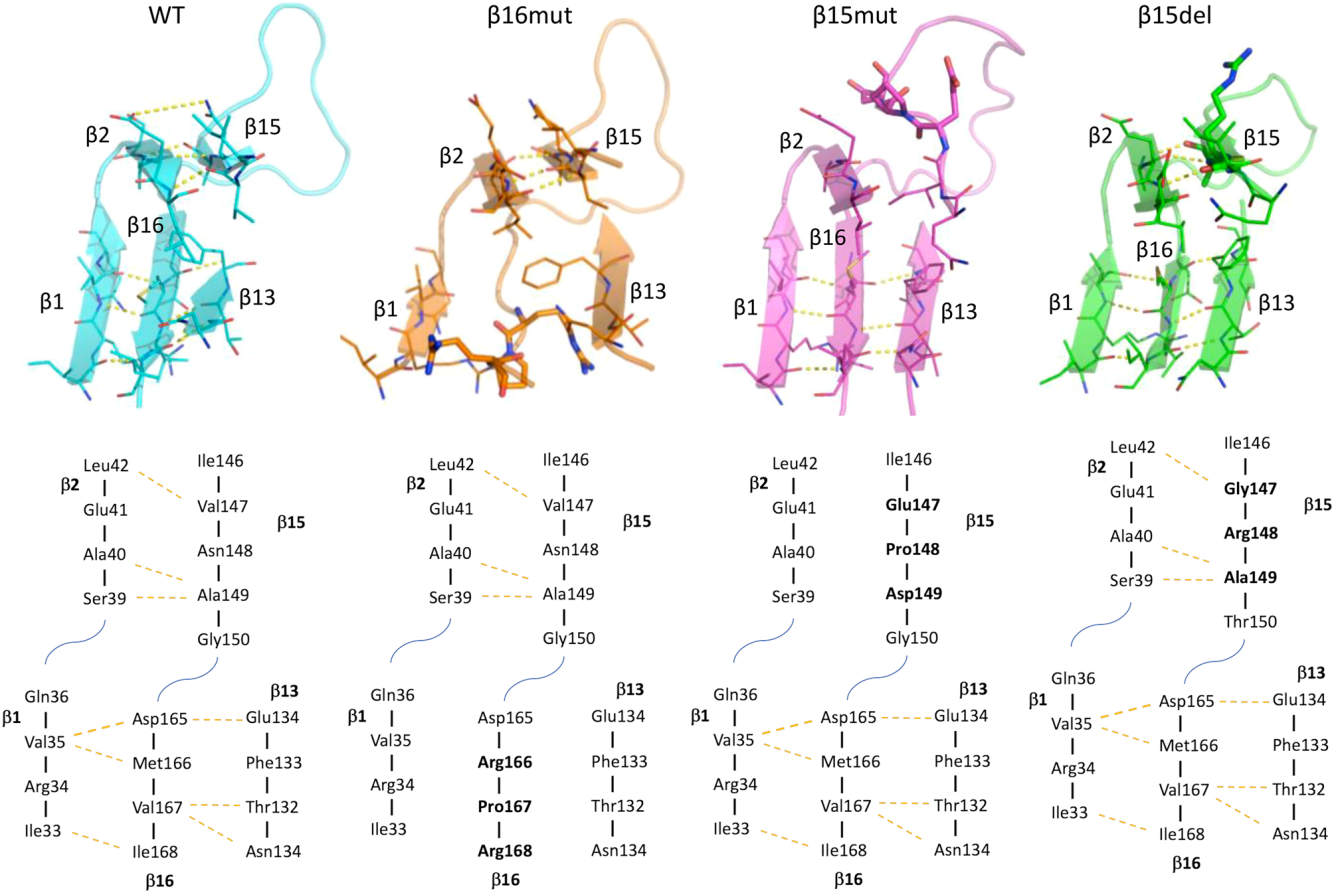
Models (upper panel) and amino acid sequences (lower panel) of the facing strands at the dimer interface for wild-type LptH and the LptH variants β16mut, β15mut and β15del. The strand secondary structures are shown as cartoon, while the mutated residues are reported as stick in the models and highlighted in bold in the amino acid sequences. H-bonds are highlighted by yellow dotted lines.

Regarding LptH_β16mut, molecular dynamics suggested that the amino acid substitution MVI^166−168^ > RPR markedly affects the secondary structure of β16. Indeed, the H-bonds between β16 and both β1 and β13, observed for the wild-type protein, disappeared at the early stage of simulation (1 ns). Moreover, the replacement of Met^166^ and Ile^168^ with the polar and bulky residue Arg could impair the hydrophobic interactions with residues of the closest strands. We also observed a partial removal of β13 from the dimerization interface (Fig. 6). The observed rearrangements and the loss of β16 polar and non-polar contacts might reasonably account for the unbinding of the dimers.

The trajectory analysis suggested that the mutations introduced in the LptH variants β15mut and β15del could be less detrimental for the dimer stability. The amino acid substitution VNA^147−149^ > EPD in LptH_β15mut was predicted to disrupt the secondary structure of β15 and, accordingly, the H-bonds with β2 disappeared during the simulation. However, the rearrangement of β15 moved the Ile^146^ side chain toward the hydrophobic groove. Thus, Ile^146^ and also Gln^145^ side chains could establish hydrophobic interactions with the residues of the closest stands, differently from what observed for the wild-type protein. The LptH_β15mut dimer appeared clearly less stable than the wild-type dimer, because of the loss of the H-bond network, but more stable than the LptH_β16mut dimer, mainly due to the larger number of contacts retained during the simulation (Fig. 6). Notably, trajectory analysis of the LptH_β15del dimer predicted that the deletion of the VNA triplet could only marginally affect the secondary structure and the contacts of β15. Indeed, the site occupied by the deleted residues was filled by the following amino acids (GRA), which have similar chemical properties, and, accordingly, the H-bond network with both β2 and β14 was maintained during the whole simulation (100 ns) (Fig. 6). Also, the hydrophobic interactions were retained, although slightly weakened by the substitution of Val^147^ with Gly. Furthermore, the deletion did not affect the adjacent strand β16, mainly because of the presence of a long loop between β15 and β16 that can change conformation, thus minimizing the effect of the deletion on the conformation of β16 (Fig. 6).

## Discussion

A wealthy of information about the working mechanism of the Lpt system has been obtained from studies performed in *E. coli.* A model has been proposed in which LPS is sequentially pushed from the IM complex LptB_2_FG to the OM translocon LptDE through a periplasmic hydrophobic groove formed by the periplasmic domains of LptC and LptD connected through the periplasmic protein LptA^3,4,8^. While this periplasmic bridge was thought to involve an LptA multimer^10–12^, a recent study showed that an LptA monomeric variant lacking the C-terminal region is still able to support *E. coli* growth, leading to propose that LptA oligomerization might not be an essential requirement for LPS transport^13^. Recent studies revealed that also LptH, the *P. aeruginosa* orthologue of *E. coli* LptA, mainly exists as a dimer in solution in vitro^14,15^, and conditional mutagenesis confirmed that this protein is essential for LPS transport and cell viability also in *P*. *aeruginosa*^16,22^. However, the relevance of the dimerization sites for the in vivo functionality of *P. aeruginosa* LptH was not yet investigated.

In this study, we used in silico modelling to predict the amino acid residues likely responsible for LptH dimerization, and genetic and biochemical assays to confirm their role in oligomerization as well as their effect on LptH functional properties in vivo. Our bioinformatic analysis confirmed that, in line with the head-to-tail model proposed for the *E. coli* LptA protein^10^, LptH dimerization involves contacts of β1 and β2 strands in the head monomer with β15 and β16 strands in the tail monomer. Our analysis also revealed a potential role of the β13 strand of the tail monomer in stabilizing the monomer–monomer interaction (Fig. 1C). Ectopic expression in an arabinose-dependent *lptH* conditional mutant of IPTG-inducible *lptH* variants, carrying either a 3-amino acid deletion or a 3-amino acid substitution in each β-strand of interest, revealed that all the in silico-identified sites are crucial for LptH activity in vivo*.* Indeed, we demonstrated that the residual growth observed for the recombinant strains expressing the LptH variants (Fig. 2) was due to the presence of revertant clones in which the arabinose-dependent wild-type copy of *lptH* inserted in the genome recombined with the mutant allele carried by the complementing plasmid (Figs. 3 and S3). Accordingly, for most protein variants, residual growth and appearance of revertant clones were abrogated upon deletion of the *recA* gene (Fig. 4), essential for homologous recombination^21^.

An exception was the LptH_β15del variant, that was found to support *P. aeruginosa* growth at levels almost comparable to the wild-type protein (Figs. 3, 4 and S3), and had no relevant defects in protein folding, stability and dimerization in in vitro assays (Fig. 5). This is in line with bioinformatics predictions suggesting that LptH_β15del has dimerization properties comparable to wild-type LptH (Fig. 6). This, however, does not imply that β15 is not important for LptH functionality, as we found that the complementary mutant variant LptH_β15mut, that carries a 3-amino acid substitution in β15 (Fig. 1C and Table 1), did not support bacterial growth (Figs. 4 and S3), even if it was able to dimerize in vitro (Fig. 5). In silico modelling suggests that the β15 conformation is likely destabilized in LptH_β15mut (Fig. 6), leading to hypothesize that, while this β-strand may not be essential for dimerization, it could be important for other LptH functions. By analogy with the current *E. coli* model, the C-terminal region of LptH should mediate the interaction with LptD^7^, so it could be speculated that β15 might be involved in this process. We cannot however exclude that it could also be important for LPS binding and/or translocation, even if the corresponding β-strand in the *E. coli* orthologue LptA harbors any of the residues found to be involved in LPS binding^9,14,23,24^. The finding that the β15 strand might not be essential for LptH dimerization highlights a first potential peculiarity of *P. aeruginosa* LptH with respect to *E. coli* LptA, in which substitutions of some β15 residues were found to abolish oligomerization in vitro^25^.

Concerning the other LptH variants, we confirmed that, besides being nonfunctional in vivo, they are also impaired in in vitro oligomerization (Fig. 5). However, some of them also showed significantly reduced stability in vitro (Fig. 5), thus hampering to evaluate the specific contribution of dimerization to protein functionality. In contrast, both variants in the β16 strand (LptH_β16del and LptH_β16mut) showed folding and stability properties comparable to the wild-type protein, but impaired dimerization (Fig. 5), indicating that this strand is specifically involved in protein oligomerization. Moreover, complementation assays showed that β16 is also essential for LptH functionality in vivo (Figs. 2, 4 and S3). Notably, we did not undeniably prove that the essentiality of the β16 strand only depends on its importance for LptH dimerization, as the effect of the introduced mutations on LPS binding and/or on the interaction with other Lpt components has not been investigated in this work. However, the crucial role of β16 for LptH functioning highlights a second relevant difference between LptH and its *E. coli* counterpart LptA. As anticipated above, it has recently been reported that a monomeric LptA variant lacking 25 amino acids at the C-terminus, including the β16 strand, can partially support *E. coli* growth^13^, strongly suggesting that this region is important for oligomerization but dispensable for in vivo LptA activity. Since the C-terminal regions of both LptA and LptH are also involved in the interaction with LptD^7^, we cannot exclude that the in vivo essentiality of the LptH β16 strand might rely on its role in LptD binding rather than (or besides) its relevance for LptH oligomerization. Further studies are clearly needed to address this issue and to verify the role of the LptH β16 strand in LptD binding and, thus, in the formation of a stable periplasmic Lpt bridge. Nevertheless, our preliminary observations suggest that the interaction between LptD and LptA/LptH could have different requirement(s) in *E. coli* and *P. aeruginosa*.

Another interesting finding is that the expression of the non-functional LptH variants investigated in this work had no inhibitory effects on bacterial growth in the presence of wild-type LptH (Figure S2), implying that they do not affect the transport of LPS and, thus, the assembly of a functional Lpt complex. While some LptH variants appeared misfolded and/or unstable and, thus, could be unable to interact with the LptH interactors LptC and/or LptD in vivo, others displayed proper folding and stability, at least in vitro (Fig. 5). Considering that each LptH variant is mutated in either the N- or C-terminal region, corresponding to the LptC and LptD binding sites, respectively^5,7^, this observation raises the possibility that the *P. aeruginosa* Lpt complex could discriminate between functional and non-functional LptH molecules. This is suggestive of a highly-regulated assembly process, as previously documented for the *E. coli* Lpt system^7^.

Overall, this study provides an initial structure-driven functional characterization of *P. aeruginosa* LptH. Besides the identification of protein residues important for LptH functionality, our results also highlight some important differences between *P. aeruginosa* LptH and the orthologous protein LptA of *E. coli*. This adds to the previously described peculiarities of the *P. aeruginosa* LptDE translocon, that differs from the *E. coli* counterpart for the presence of an additional domain of unknown function at the LptD N-terminus^14^, a larger lumen volume^26^, and the specific role of LptE, that is important as LptD chaperone and plug but is not directly involved in LPS transport^22^. The unique features of the *P. aeruginosa* LptD periplasmic domain have been proposed to justify the anti-pseudomonads specificity of recently identified peptidomimetics targeting LPS transport through interaction with LptD^27–29^. Since the cell envelope biogenesis pathways are nowadays considered attractive targets for novel antibacterial drugs^29–31^, this emphasizes the potential impact of investigating the conserved and divergent aspects of these systems in different human pathogens. This information could indeed ultimately drive the rational design of new narrow- or broad-spectrum antibacterial agents.

## Methods

### Bacterial strains and growth conditions

Bacterial strains used in this work are listed in Table 1. *E. coli* and *P. aeruginosa* were routinely grown in Lysogeny Broth, Lennox formulation (LB, Acumedia) for general genetic procedures and protein expression assays, and in Mueller–Hinton (MH) broth (Acumedia) for growth assays. When required, antibiotics were added at the following concentrations for *E. coli*, while the concentrations used for *P. aeruginosa* are shown in brackets: ampicillin, 100 μg/mL; tetracycline, 12 μg/mL (50–100 μg/mL); nalidixic acid, 15 µg/mL; chloramphenicol, 30 μg/mL (375 μg/mL); kanamycin, 25–50 μg/mL.

### Molecular modeling

All molecular modeling studies were performed on a MacPro dual 2.66 GHz Xeon running Ubuntu 14 LTS. The images in the manuscript were created with PyMOL. (PyMOL version 1.7.0.0 DeLano Scientific LLC: San Carlos, CA.) The LptH monomer structure (pdb code 4UU4) was downloaded from the PDB web site (https://www.rcsb.org)^14^. The dimer was obtained by homology model, using *E. coli* LptA (pdb code 2R19) as reference structure^10^. Proteins were prepared by the Protein Preparation Wizard of the Maestro suite^32^. The mutated forms of the dimers were designed using the single-residue mutation option of Maestro GUI. The mutations were introduced just in the strands located in the interface between the monomers. Then, each system was minimized by OLPS3 force field (Small-Molecule Drug Discovery Suite 2018-1, Schrödinger) with 2,500 maximum iterations and 0.05 as the convergence threshold.

Molecular dynamics was performed with the Amber 12 suite^33^. The minimized structure was solvated in a periodic octahedron simulation box using TIP3P water molecules, providing a minimum of 10 Å of water between the protein surface and any periodic box edge. Ions were added to neutralize the charge of the total system. The water molecules and ions were energy-minimized, keeping the coordinates of the dimers fixed (1,000 cycle), and then the whole system was minimized (2,500 cycle). Following minimization, the entire system was heated to 298 K (20 ps). The production (100 ns) simulation was conducted at 298 K with constant pressure and periodic boundary condition. Shake bond length condition was used (ntc = 2). Production was carried out on GeForce gtx780 gpu. Trajectories analysis were carried out by the CPPTRAJ program^34^ H-bonds formation rates and H-bonds distances were computed by CPPTRAJ and Chimera, respectively^35^.

### Growth assays

Growth assays in liquid media were performed in MH broth at 37 °C in microtiter plates (200 µL per well) at 200 rpm both in the absence or in the presence of arabinose and/or IPTG at the indicated concentrations. Strains of interest were cultured overnight at 37 °C and refreshed 1:2,000 (about 2 × 10^6^ cells/mL) in fresh medium. When indicated, overnight cultures were normalized to an optical density at 600 nm (OD_600_) = 1 in saline, serially diluted 1:10 in saline and 5 μL of 10^−5^ dilution (containing about 50–100 cells) were inoculated in fresh medium. Bacterial growth was measured as the OD_600_ of the bacterial cultures in a Victor plate reader (Wallac).

Growth assays on solid media were performed by plating 5 μL of serial ten-fold dilutions from bacterial suspensions in saline normalized to an OD_600_ = 1 (from late-exponential cultures grown in the presence of arabinose) on MH solidified with 1.5% agar. When required, 0.5% arabinose or 0.5 mM IPTG was added to the medium. Plating efficiency was determined as the ratio between the number of colony forming units (CFU)/mL obtained under testing condition(s) and the CFU/mL obtained under permissive condition (presence of arabinose).

### Construction of plasmids and mutant strains

*E. coli* was used as host for recombinant DNA manipulations. PCR primers and restriction enzymes used for cloning are listed in Table S2, while the plasmids used or generated in this work are described in Table 1. All constructs were verified by DNA sequencing.

The construct pBS *lptH* was generated by cloning the *lptH* coding sequence together with its putative RBS into the sequencing plasmid pBluescript II (pBS) by EcoRI/XhoI digestion. This construct was then used as template for PCR-mediated site-specific mutagenesis of the *lptH* gene using the "Q5 Site-Directed Mutagenesis" kit (New England BioLabs), according to manufacturer’s instructions, and primer pairs specifically designed to introduce 9-bp deletions or 9-bp substitutions in the regions of interest (Table S2). Samples were then used to transform high efficiency *E. coli* NEB 5-alpha competent cells provided by the kit. The positive clones (containing the mutated *lptH* genes) were screened by colony PCR, using a “CHECK” reverse primer (specific for each mutation) coupled with the forward primer *lptH*_FW, and confirmed by plasmid extraction and sequencing of the entire *lptH* coding sequence. The *lptH* gene and its mutant variants were then excised from the corresponding pBS constructs by EcoRI/XhoI digestion and subcloned into the IPTG-inducible shuttle vector pME6032^36^ using the same enzymes. The resulting constructs were individually introduced into the *P. aeruginosa* *lptH* conditional mutant PAO1 *araC*P_BAD_*lptH* Δ*lptH* by transformation.

For expression of LptH6His and its mutant variants in *E. coli*, the wild-type and mutant genes, lacking the signal peptide-encoding region, were amplified with primers *lptH*_pET28b_FW and *lptH*_pET28b_RV, using the corresponding pBS constructs as templates, and cloned into pET28b using NdeI/HindIII restriction sites (Table S2). The resulting constructs were introduced into *E. coli* BL21 (DE3) by transformation.

Unmarked in-frame deletion mutants in *recA* were constructed by suicide plasmid insertion mutagenesis. The construct for mutagenesis was generated by directionally cloning two PCR-amplified DNA fragments of *ca.* 500 bp, encompassing the regions upstream and downstream of the sequence to be deleted, in the *sacB*-containing suicide vector pDM4^37^, generating the deletion mutagenesis vector pDM4Δ*recA.* This plasmid was conjugally transferred from *E. coli* S17.1 λ*pir* into *P. aeruginosa* PAO1 or the *lptH* conditional mutant, and transconjugants were selected on LB agar plates containing 15 μg/mL nalidixic acid and 350 μg/mL chloramphenicol. Resolution of merodiploids was obtained by plating onto LB agar plates containing 10% sucrose^38^. Chloramphenicol-sensitive clones were screened by PCR to identify deletion mutants, which were then verified by DNA sequencing.

### Protein expression and purification

Wild-type LptH and its variants were expressed as N-terminally His-tagged proteins using the pET28b vector and purified using the following protocol. *E. coli* BL21(DE3) cells, transformed with the expression vectors, were grown in LB medium supplemented with 25 μg/mL kanamycin at 37 °C to OD_600_ ~ 0.8. Protein expression was induced by the addition of 0.5 mM IPTG and cells were cultured at 37 °C for other 2 h. Cells were harvested by centrifugation, washed in 20 mM Tris–HCl, pH 8.0, resuspended in 20 mM Tris–HCl, pH 8.0, 250 mM NaCl, 20 mM imidazole containing a Protease Inhibitor Cocktail Tablet (Roche), and lysed by sonication in ice. After centrifugation to remove cell debris, the soluble fraction was loaded into a HisTrap FF column (GE Healthcare) equilibrated with 20 mM Tris–HCl, pH 8.0, 250 mM NaCl, 20 mM imidazole (binding buffer). Proteins were eluted in the fraction containing binding buffer supplemented with 200 and/or 300 mM imidazole and the purity was verified by SDS-PAGE (Figure S4). The recombinant protein was then concentrated to a final volume of 2.5 mL using an Amicon Ultra-15 (Millipore) and then buffer exchanged in a PD-10 pre-packed column (GE Healthcare) to 20 mM Tris–HCl, pH 8.0, 250 mM NaCl. Protein concentration was determined spectrophotometrically at 280 nm by using the calculated extinction coefficient of each protein variant.

### Circular dichroism (CD) spectroscopy

CD experiments were performed using a Jasco J710 instrument (Jasco Inc., Easton, MD, USA) equipped with a Peltier apparatus for temperature control. Spectra were collected in the far-UV region (195–250 nm) using a quartz cell with 1 mm optical path length at a scanning speed of 100 nm/min. Static spectra of LptH and its variants (15 μM) in 50 mM sodium phosphate buffer pH 7.2, 250 mM NaCl are the average of three scans. Thermal denaturation experiments were performed by monitoring the CD signal at 220 nm as a function of temperature (1 °C/min thermal ramp, from 25 °C to 80 °C). Data were fitted to a sigmoid function.

### HPLC analysis

The oligomerization states of wild-type LptH and its variant proteins were analysed by size-exclusion chromatography. 100 μL of sample (protein concentration: 2.0 mg/mL for wild-type LptH; 1.4 mg/mL for LptH_β16mut and LptH_β16del; 1.9 mg/mL for LptH_β13mut; 1.3 mg/mL for LptH_β13del; 1.6 mg/mL for LptH_β1mut; 1.8 mg/mL for LptH_β1del; 1.4 mg/mL for LptH_β15mut; 0.9 mg/mL for LptH_β15del; 1.5 mg/mL for LptH_β2del) were loaded into a TSK-GEL G3000PW_XL_ column (Tosoh Bioscience) equilibrated with 20 mM Tris–HCl, pH 8.0, 250 mM NaCl and connected to an HLPC AZURA system (KNAUER, Berlin, Germany). The flow rate was fixed at 0.7 mL/min, and detection was recorded at 280 nm. The elution volumes were: V_e_ = 7.16 mL for wild-type LptH; V_e_ = 7.39 mL for LptH_β16mut; V_e_ = 7.37 mL for LptH_β16del; V_e_ = 7.43 mL and V_e_ = 5.53 mL for LptH_β13mut; V_e_ = 7.48 mL for LptH_β13del; V_e_ = 7.33 mL and V_e_ = 5.64 mL for LptH_β1mut; V_e_ = 7.36 mL and V_e_ = 5.57 mL for LptH_β1del; V_e_ = 7.18 mL for LptH_β15mut; V_e_ = 7.21 mL for LptH_β15del; V_e_ = 5.55 mL and V_e_ = 7.39 mL for LptH_β2del. The column was calibrated using the following molecular weight standards (Sigma-Aldrich): aldolase, 155 kDa (V_e_ = 6.39 mL); conalbumin, 75 kDa (V_e_ = 6.73 mL); carbonic anhydrase, 29 kDa (V_e_ = 7.26 mL); cytochrome c, 12.4 kDa (V_e_ = 7.76 mL). Molecular weights and thus oligomerization states of wild-type LptH and variant proteins were estimated from the obtained calibration curve. Absorbance values at 280 nm were normalized between 0 and 100% and rescaled as appropriate in the different panels.

### SDS PAGE and Western blot

To assess the expression of the wild-type LptH protein and/or the LptH mutant variants in *E. coli* BL21 (DE3) or in the conditional mutant PAO1 *araC*P_BAD_*lptH* Δ*lptH*, SDS-PAGE and Western blot analyses were performed. Appropriate volumes of exponentially growing bacterial cultures were centrifuged, and pellets were suspended in SDS-PAGE loading buffer (0.25 M Tris–HCl pH 6.8, 2% SDS, 10% β-mercaptoethanol, 20% glycerol) for SDS-PAGE analysis of whole-cell extracts. Pellets from identical culture volumes were also collected to determine the cellular protein concentration of each sample by using the DC protein assay kit (Bio-Rad) with bovine serum albumin as the standard. Volumes of SDS-PAGE samples corresponding to 20 μg of protein were loaded onto the gels. Proteins resolved by SDS-PAGE were electrotransferred onto a nitrocellulose filter (Hybond-C extra; Amersham) and probed for LptH with custom rabbit polyclonal antibodies or for the 6His tag with a mouse polyclonal antibody (Sigma-Aldrich). Anti-LptH antibodies were generated at GenScript (https://www.genscript.com/custom-polyclonal-antibody-production-services.html) with a keyhole limpet hemocyanin-conjugated peptide as the antigen (LptH epitope, GRATGSQVTSPRPR), which was selected with the OptimumAntigen design tool (GenScript). An anti-LptC polyclonal antibody^17^ was used to normalize *P. aeruginosa* protein samples for the housekeeping protein LptC. Goat anti-rabbit or rabbit anti-mouse IgG horseradish peroxidase-conjugated secondary antibodies (Sigma-Aldrich) were used. Filters were developed with ECL chemiluminescent reagents (Amersham), visualized on a ChemiDoc XRS + system and processed with the Image Lab 3.0 software (Bio-Rad). When required, changes to brightness and contrast were applied equally across the entire images.

## Supplementary information

Supplementary information
