# Supplementary material for: Mutational analysis of the essential lipopolysaccharide-transport protein LptH of Pseudomonas aeruginosa to uncover critical oligomerization sites
Source: Sci Rep. 2020 Jul 9;10:11276. doi: 10.1038/s41598-020-68054-7 (PMC7347655; doi:10.1038/s41598-020-68054-7)
Supplement: Supplementary file 1 — Supplementary information [file 41598_2020_68054_MOESM1_ESM.pdf]

SUPPLEMENTARY INFORMATION

Mutational analysis of the essential lipopolysaccharide-transport protein LptH of *Pseudomonas aeruginosa* to uncover critical oligomerization sites

Romina Scala, Adele Di Matteo, Antonio Coluccia, Alessandra Lo Sciuto, Luca Federici, Carlo Travaglini-Allocatelli, Paolo Visca, Romano Silvestri, Francesco Imperi

Table S1. Frequency of spontaneous mutants resistant to ofloxacin or gentamicin in the *P. aeruginosa lptH* conditional mutant carrying the indicated constructs.

**Construct**	**Frequency of spontaneous resistant mutants**[a]	
	**Ofloxacin**	**Gentamicin**
pME *lptH*	3.3×10^{-7}	1.9×10^{-7}
pME *lptH*_β16mut	1.7×10^{-7}	2.6×10^{-7}
pME *lptH*_β13mut	3.0×10^{-7}	1.6×10^{-7}

[a] Calculated from three independent assays.

Table S2. Primers used in this work.

Primer	Sequence (5'-3')[a]	Restriction sites[b]	Application
*lptH*_FW	cggaattcATGCACCTGCTGTCCAACG	EcoRI	Generation of pBS
*lptH*_RV	ccgctcgagtcaatggtgatggtgatggtgCTGGGCC TTTTTCTTCGGC	XhoI	*lptH*
*lptH*_β16del_FW	CAGCCGAAGAAAAAGGCCCAG		Generation of β
*lptH*_β16del_RV	GTCGATGCGTGGGCGCGG		*lptH*_del1
*lptH*_β16mut_FW	acgaCAGCCGAAGAAAAAGGCCCAG		Generation of pBS
*lptH*_β16mut_RV	ggtcgGTCGATGCGTGGGCGCGG		*lptH*_mut1
*lptH*_β13del_FW	GAAGGCGAGAAGATCGTCTAC		Generation of pBS
*lptH*_β13del_RV	GCCTTCCTGGATCACCTTGG		*lptH*_del2
*lptH*_β13mut_FW	agctGAAGGCGAGAAGATCGTCTAC		Generation of pBS
*lptH*_β13mut_RV	ggggcGCCTTCCTGGATCACCTTGG		*lptH*_mut2
*lptH*_β1del_FW	CAGGCCGACAGCGCCGAA		Generation of pBS
*lptH*_β1del_RV	CGGTTGTTCGCGGTCCGAAG		*lptH*_del3
*lptH*_β1mut_FW	tgatCAGGCCGACAGCGCCGAA		Generation of pBS
*lptH*_β1mut_RV	ggttcCGGTTGTTCGCGGTCCGAAG		*lptH*_mut3
*lptH*_β2del_FW	GACGACAAGCAGGGCGTCG		Generation of pBS
*lptH*_β2del_RV	GCTGTCGGCCTGGACGCG		*lptH*_del4
*lptH*_β2mut_FW	tcgcGACGACAAGCAGGGCGTCG		Generation of pBS
*lptH*_β2mut_RV	ggacgGCTGTCGGCCTGGACGCG		*lptH*_mut4
*lptH*_β15del_FW	GGTCGTGCCACCGGCTCC		Generation of pBS
*lptH*_β15del_RV	GATCTGGCGCTGGGTGTCG		*lptH*_del5
*lptH*_β15mut_FW	tgatGGTCGTGCCACCGGCTCC		Generation of pBS
*lptH*_β15mut_RV	ggttcGATCTGGCGCTGGGTGTCG		*lptH*_mut5
*lptH*_β16del_CHECK	CTTCGGCTGGTCGATGCG		Colony PCR
*lptH*_β16mut_CHECK	CGGCTGTCGTGGTCGGTC		screening of pBS
*lptH*_β13del_CHECK	TCGCCTTCGCCTTCCTGG		constructs with the
*lptH*_β13mut_CHECK	CTCGCCTTCAGCTGGGGC		different *lptH*
*lptH*_β1del_CHECK	GTCGGCCTGCGGTTGTTC		variants (each
*lptH*_β1mut_CHECK	TGTCGGCCTGATCAGGTTC		reverse primer was
*lptH*_β2del_CHECK	GCTTGTCGTCGCTGTCGG		coupled with
*lptH*_β15del_CHECK	TGGCACGACCGATCTGGC		*lptH*_FW)
*lptH*_β15mut_CHECK	TGGCACGACCATCAGGTTC		
*lptH*_pET28b_FW	ggaattccataTGCCTTCGGACCGCGAAC	NdeI	Generation of
*lptH*_pET28b_RV	cccaagctttcaCTGGGCCTTTTTCTTCGGC	HindIII	pET28b *lptH* and mutant constructs
recA mut_UP_FW	ccgcTCGAGGCTGGCTACGTGAC	XhoI	Generation of
recA mut_UP_RV	cgggatCCGAATTGGCGTTCGATCTG	BamHI	pDM4 Δ*recA*
recA mut_DOWN_FW	cgggatCCGAAGAAGTGGCTGACGC	BamHI	
recA mut_DOWN_RV	gctctaGAAAACTGGGACTGAAGCTG	XbaI	
M13_FW	GTTTTCCCAGTCACGAC		DNA sequencing
M13_RV	AACAGCTATGACCATG		
T7_FW	TAATACGACTCACTATAGGG		

[a] Lowercase letters indicate the region of the primer that does not anneal to the template.
[b] The restriction site used for cloning is underlined in the primer sequence.

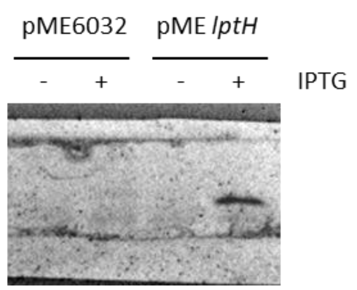

Figure S1. Detection of LptH in the *P. aeruginosa lptH* conditional mutant carrying the empty vector pME6032 or pME *lptH* cultured in MH containing 0.5% arabinose and supplemented (+) or not (-) with 0.5 mM IPTG, by Western blotting of whole-cell lysates (20 μg total proteins) with an anti-LptH polyclonal antibody. The image is representative of two independent experiments which gave similar results.

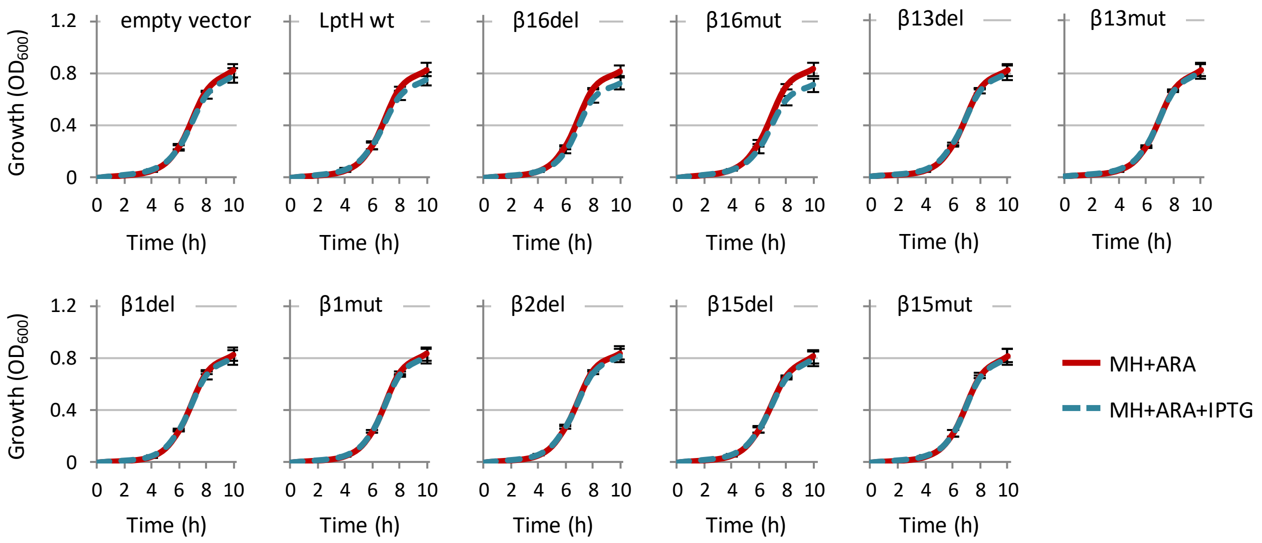

Figure S2. Growth curves of the *P. aeruginosa lptH* conditional mutant carrying the empty plasmid pME6032, pME6032 with wild-type *lptH* or pME6032 with different mutant variants in MH at 37°C in microtiter plates in the presence of 0.5% arabinose (+ ARA; red lines) or 0.5% arabinose and 0.5 mM IPTG (+ ARA + IPTG; dashed blue lines). Growth was measured as OD_{600}. Results are the mean (± SD) of two independent experiments, each performed in triplicate.

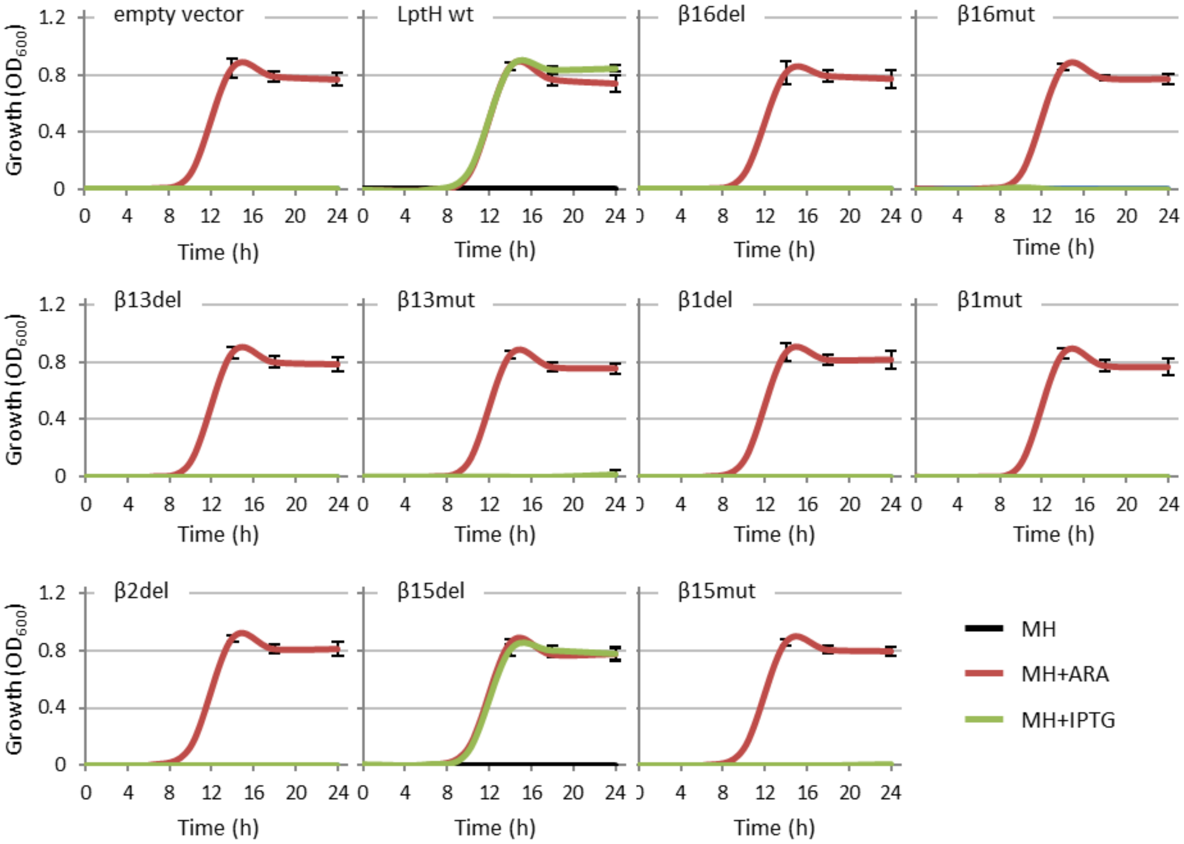

Figure S3. Growth curves of the *P. aeruginosa lptH* conditional mutant carrying the empty plasmid pME6032, pME6032 with wild-type *lptH* or pME6032 with different mutant variants inoculated at a cell density of ca. 50-100 cells/mL in MH at 37°C in microtiter plates in the absence (black lines) or in the presence of 0.5% arabinose (+ ARA; red lines) or 0.5 mM IPTG (+ IPTG; green lines). Growth was measured as OD_{600}. Results are the mean (± SD) of three independent experiments, each performed in quadruplicate.

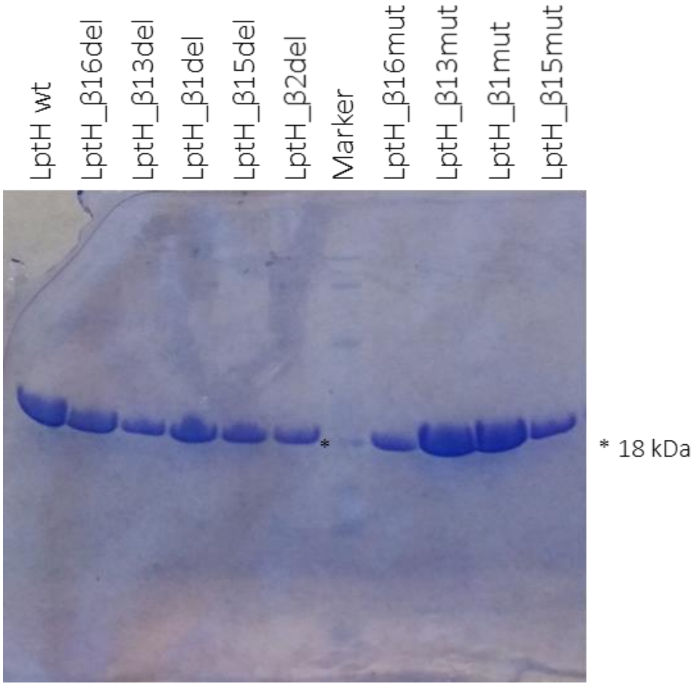

Figure S4. Coomassie-stained SDS-PAGE gel showing the proteins purified in this work.

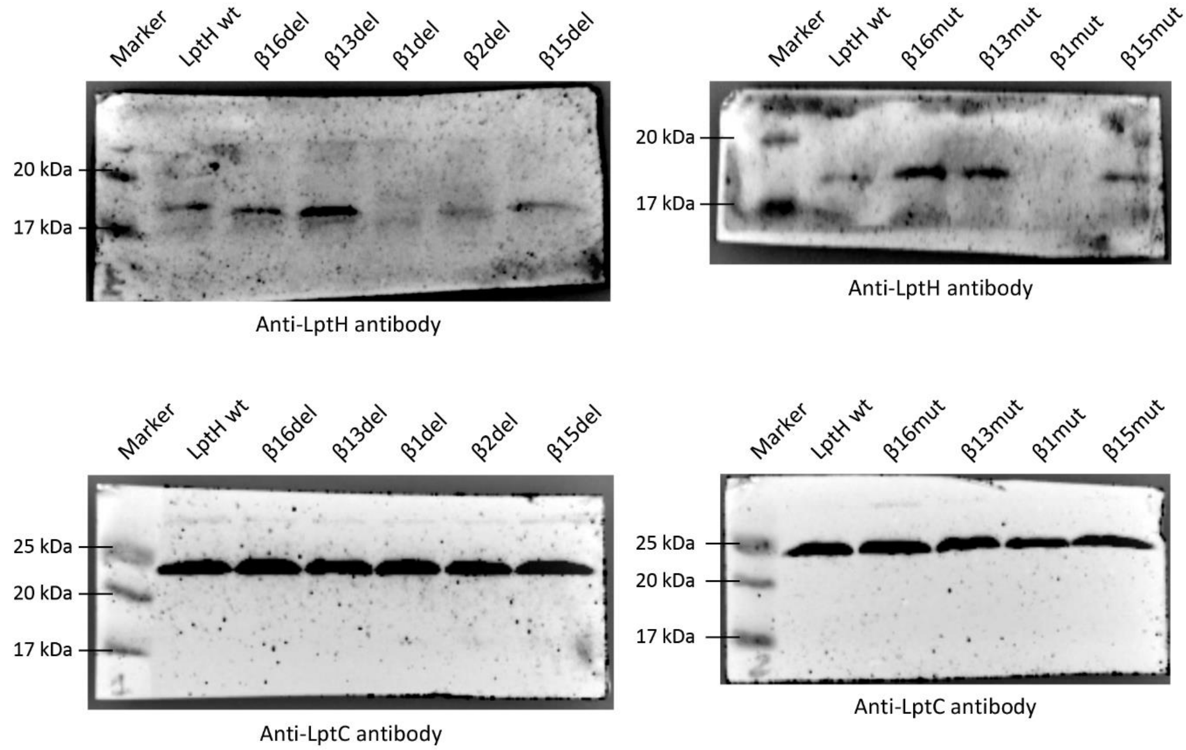

Figure S5. Full-length blots for the cropped images shown in Figure 2B.
